# [Corrigendum] DJ‑1 is involved in the peritoneal metastasis of gastric cancer through activation of the Akt signaling pathway

**DOI:** 10.3892/or.2024.8797

**Published:** 2024-08-14

**Authors:** Zheng-Ming Zhu, Zheng-Rong Li, Yan Huang, Hai-Hong Yu, Xiao-Shan Huang, Yu-Feng Yan, Jiang-Hua Shao, He-Ping Chen

Oncol Rep 31: 1489–1497, 2014; DOI: 10.3892/or.2013.2961

Subsequently to the publication of the above paper, an interested reader drew to the authors' attention that the western blot data shown for the MMP-9 experiment in Fig. 4 on p. 1493 were strikingly similar to the western blots shown for the total-Akt experiments in Fig. 6 on p. 1494.

After having re-examined their original data files, the authors realized that Fig. 6 had been inadvertently assembled incorrectly. The revised version of Fig. 6, containing the correct data for the total-Akt experiments, is shown below. Note that the corrections made to this figure do not affect the overall conclusions reported in the paper. The authors are grateful to the Editor of *Oncology Reports* for allowing them the opportunity to publish this Corrigendum, and apologize to the readership for any inconvenience caused.

## Figures and Tables

**Figure 6.**
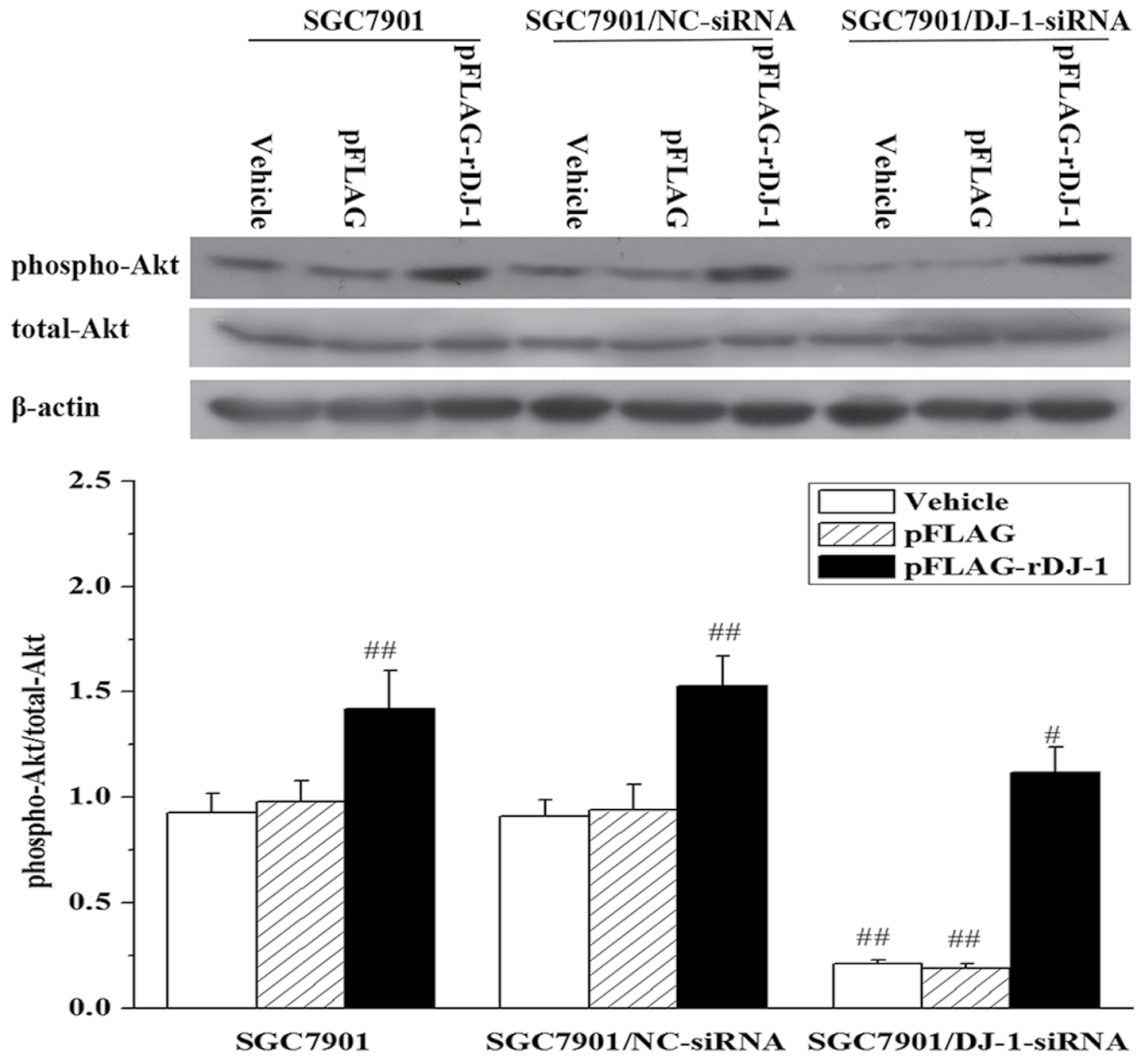
DJ-1 regulates the phosphorylation of Akt. SGC7901, SGC7901/NC-siRNA and SGC7901/DJ-1-siRNA cells were transfected with or without pFLAG or pFLAG-rDJ-1. Twenty-four hours later, the expression of phospho-Akt and total-Akt proteins was analyzed by western blot analysis. β-actin was used as an internal control. A representative blot of each experiment is shown with the densitometric analysis corresponding to the mean ± SD of four independent experiments. ^#^P<0.05, ^##^P<0.01 vs. SGC7901 + Vehicle.

